# How unique is the tiger beetle fauna (Coleoptera, Cicindelidae) of the Balkan Peninsula?

**DOI:** 10.3897/zookeys.100.1542

**Published:** 2011-05-20

**Authors:** Radomir Jaskuła

**Affiliations:** Department of Invertebrate Zoology and Hydrobiology, University of Łódź, Banacha 12/16, 90-237 Łódź, Poland

**Keywords:** Balkan Peninsula, biodiversity, distribution, Europe, zoogeography

## Abstract

The tiger beetle fauna of the Balkan Peninsula is one of the richest in Europe and includes 19 species or 41% of the European tiger beetle fauna. Assembled by their biogeographical origins, the Balkan tiger beetle species fall into 14 different groups that include, Mediterranean, Middle Oriental, Central Asiatic, Euro-Siberian, South and East European, Pannonian-Sarmatian, West Palaearctic, Turano-European and Afrotropico Indo-Mediterranean species. The Mediterranean Sclerophyl and the Pontian Steppe are the Balkan biogeographical provinces with the highest species richness, while the Balkan Highlands has the lowest Cicindelidae diversity. Most species are restricted to single habitat types in lowland areas of the Balkan Peninsula and only *Calomera aulica aulica* and *Calomera littoralis nemoralis* occur in respectively 3 and 4 different types of habitat. About 60% of all Balkan Cicindelidae species are found in habitats potentially endangered by human activity.

## Introduction

Tiger beetles occur world-wide, with the exception of the polar regions and some oceanic islands (Cassola and Pearson 2000, Pearson and Vogler 2001). Detailed studies from different regions show that many species have narrow habitat specialization and occur only in one or at most in few very similar types of habitat. As a result, tiger beetles have become a significant global flagship group for beetle conservation used as biological indicators for determining global and regional patterns of biodiversity (Knisley and Hill 1992; Pearson and Cassola 1992, 2007; Carroll and Pearson 1998a, b; Andriamampianina et al 2000; Pearson and Vogler 2001; Arndt et al. 2005). In most species, adult beetles are diurnal and highly mobile, while larvae are sedentary and live in burrows constructed in the substrate where the eggs are oviposited (Pearson 1988). Both imagines and larvae are predators that prey on small invertebrates, a characteristic that makes them potentially natural biological controls of pests with an economic value (Rodriguez et al. 1998).

The Balkan Peninsula is part of the Mediterranean basin. It is one of the 25 most important world hotspot areas of biodiversity (Myers et al. 2000). Together with two other South European peninsulas, the Iberian and the Italian, the Balkans were the most important terrestrial Pleistocene glacial refugia in Europe. Phylogeographical studies on many different groups of animals and plants show that these areas are regions from which the re-colonisation of northern Europe started after the last glaciation period (Hewitt 1996, 1999; Blonden and Aronson 1999; Thompson 2005). Weiss and Ferrand (2007) suggest that high biodiversity of the South European Peninsulas, including the Balkan Peninsula, can be explained by relatively high climatic stabilization of this region as well as heterogeneous landscapes occurring in this area. Moreover, the Balkans have served as an important natural bridge for historical dispersal between Asia Minor and northern, western and central Europe (Crnobrnja-Isailovic 2007).

The first data on the tiger beetle fauna of the Balkan Peninsula were published at the end of the 19th and beginning of the 20th century (Reitter 1881; Horn and Roeschke 1891; Apfelbeck 1904–1907). Since then, more than 40 papers have been published on this topic, many of which however only describe information on a single species or present incomplete faunistic and taxonomic data. Recently more complete information on the fauna of some regions have been summarized for Bulgaria (Guéorguievand Guéorguiev1995), Montenegro (Jaskuła et al. 2005), Albania (Guéorguiev 2007; Jaskuła 2007a), Romania (Cassola and Jaskuła 2004; Jaskuła 2007b), Greece (Franzen 2005; Jaskuła et al. – in preparation) and the European part of Turkey (Cassola 1999; Avgın and Özdikmen 2007).

The aim of this paper is to summarize knowledge on the diversity of tiger beetles in the Balkan Peninsula with particular emphasis on total group diversity, zoogeographical composition, distribution, and ecological preferences of the species.

## Study area

**Figure 1.**
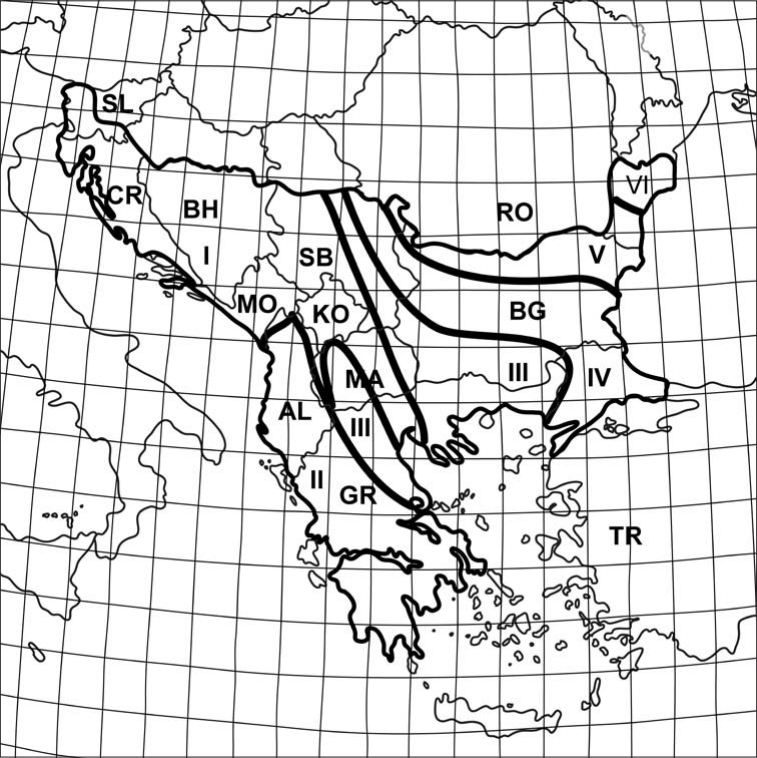
Geographical and administrative divisions of the Balkan Peninsula: **I** Dinaric region **II** Pindus region **III** Tracian-Macedonian region **IV** Balkanid region **V** Danubian plain region **VI** North-Dobroudzha region **AL** Albania **BG** Bulgaria **BH** Bosnia and Herzegovina **CR** Croatia **GR** Greece **KO** Kosovo **MA** Macedonia FYR**, MO** Montenegro **RO** Romania **SB** Serbia **SL** Slovenia **TR** Turkey.

We can define the Balkan Peninsula as a part of southeastern Europe with its northern boundary at the Danube, Sava and Kupa rivers. The rest of its margins are made up of the Black Sea in the east, the Adriatic Sea in the west, and the Mediterranean Sea (including the Aegean and Ionian seas) in the south (Fig. 1). The region has a combined area of ca. 550,000 km2, which is nearly 5% of the entire European continent. The peninsula includes twelve countries, seven of which are completely confined to the Balkan Peninsula (Albania, Bulgaria, Greece, Macedonia FYR, Montenegro, Kosovo, and Bosnia-Herzegovina), and five (Romania, Serbia, Croatia, Slovenia, and Turkey) have only a part of their territories on the peninsula.

The largest surface of the Balkan Peninsula is mountainous. Lowlands extend along the lower reaches of rivers that are grouped into three catchments draining into the Adriatic, Aegean, and Black Sea (Reed et al. 2004). Geographically this area is divided into the following main regions: Dinaric, Pindus, Tracian-Macedonic, Balkanic, Danubian plain, and North-Dobroudzha (Fig. 1).

**Figure 2.**
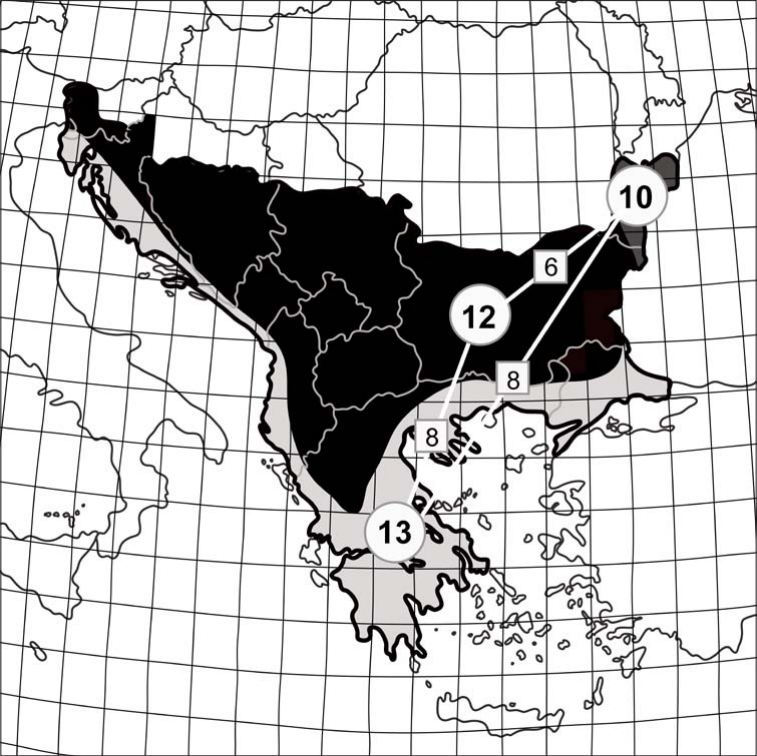
Tiger beetle faunas in the biogeographical provinces of the Balkan Peninsula (division after Udvardy 1975): light grey – Mediterranean Sclerophyl, dark grey – Pontian Steppe, black – Balkan Highlands. Numbers in the circles indicate the number of cicindelid taxa for the separate regions and the squares give the number of taxa common to the provinces shared.

According to Udvardy (1975) the Balkan Peninsula belongs to three main biogeographical provinces (Fig. 2): Mediterranean Sclerophyl – which includes European parts of Turkey, the Adriatic coast of Albania, Montenegro, Bosnia-Herzegovina, Croatia and Slovenia, and the sea coast of continental Greece; Balkan Highlands – with mountain areas of Bulgaria, Albania, Montenegro, Kosovo, Bosnia-Herzegovina, Serbia (except Voivodina), and partly also the mountains of Greece, Croatia and Slovenia, as well as the southern part of the Bulgarian Black Sea Coast; Pontian Steppe – the smallest area of the Balkans with only a small part of the northeastern Bulgarian Black Sea Coast and southeastern Romania, with its northern border at the Danube Delta.

## Material and methods

The basis for this analysis of Balkan tiger beetles comes from published literature data; such as museum collections of the Museum and the Institute of Zoology, Polish Academy of Sciences (Warsaw, Poland), Royal Belgian Institute of Natural Sciences (Brussels, Belgium), Zoological Museum (Copenhagen, Denmark), Finnish Museum of Natural History (Helsinki, Finland), University of Montenegro (Podgorica, Montenegro); and original collections made by the author in the years 2005–2009 during five scientific expeditions covering almost all Balkan countries (“I-III Amphi-Balkan expeditions” and “Ist and IIIrd TB-Quest expeditions”).

Tiger beetle species richness and distribution of taxa were analysed based on squares of 1o latitude and longitude. In each square the number of all species recorded was summarized. Similarities among tiger beetle fauna between geographical units were measured using the Bray-Curtis index for presence*/*absence data (Primer v.2.0). Jaccard’s (1902) index was used to present the degree of dissimilarity between zoogeographic regions distinguished by Udvardy (1975):

R= 100c/a+b-c

where: a = number of species in the richest fauna; b = number of species in the poorest fauna, c = number of species comon to both faunas.

Chorotypes follow Vigna Taglianti et al. (1999).

## Results

### Diversity of tiger beetles in the Balkan Peninsula

According to Putchkov and Matalin (2003), López et al. (2006) and Fauna Europea Web Service (2004) 49 tiger beetle species occur in Europe. Of these, 19 species have been found in the area of the Balkan Peninsula (Table 1), or 39% of all European tiger beetle species. This number increases to 41% if three species known only from Mediterranean islands of Europe are excluded (*Cephalota tibialis* – Cyprus, *Calomera lunulata* – Sicily, *Habrodera nilotica* – Canary Islands). The Balkan species belong to five genera (55.5% of European fauna) including: *Myriochila* (1 species, 50% of European species), *Cephalota* (4 species, 33%), *Calomera* (4 species, 57%), *Cylindera* (4 species, 57%), and *Cicindela* (7 species, 41%). Only four European genera – *Megacephala*, *Lophyra*, *Cassolaia* and *Habrodera* do not occur in this area. Two taxa (*Cicindela campestris oliviera* and *Cicindela monticola albanica*) are endemic to this area. Additionally, for eleven species the Balkan Peninsula is the only place in Europe where they occur (having also distributions outside Europe).

**Table 1.** Comparison of area and tiger beetle species richness of some European regions [based on Putchkov and Matalin (2003) and Fauna Europea Web Service (2004)].

*Region*	*Area(km2)*	*Number of species*	*Species density(species number/1000 km2)*
Balkan Peninsula	550 000	19	0.034
Iberian Peninsula	580 000	19	0.033
Italian Peninsula	150 000	13	0.086
Scandinavian Peninsula	800 000	5	0.006
France (mainland)	675 000	14	0.021
Ukraine	603 700	19	0.031
Russia (European part)	4 268 850	23	0.005

The number of Balkan tiger beetle species is high compared with the number noted from other European regions with similar sized areas, especially north of the Balkan Peninsula (Table 1). Moreover, the diversity of the tiger beetle fauna in the studied area is similar to the fauna known from the entire territory of the European part of Russia. Among European regions with a similar area, only the Iberian Peninsula and the Ukraine exhibit similar numbers of tiger beetle species.

Balkan Cicindelidae belong to 14 different groups according to their geographical origin (Vigna Taglianti et al. 1999, Table 2). Except Balkan endemics and Mediterranean species, representatives of Middle Oriental, Central Asiatic, Euro-Siberian, South and East European, Pannonian-Sarmatian, West Palaearctic, Turano-European, or even Afrotropico Indo-Mediterranean taxa can be found in this area.

**Table 2.** Chorotypes of Balkan tiger beetles (after Vigna Taglianti et al. 1999).

*Balkan endemics*	*Cicindela campestris oliviera*, *Cicindela monticola albanica*
Mediterranean	*Calomera littoralis nemoralis*, *Cephalota circumdata circumdata*, *Calomera aulica aulica*
East Mediterranean	*Calomera concolor concolor*
West Mediterranean	*Cylindera trisignata trisignata*
Middle Oriental	*Calomera fischeri fischeri*
Central Asiatic	*Cephalota chiloleuca*, *Cylindera contorta contorta*
Northeast Mediterranean (Aegean)	*Cephalota turcica*, *Cylindera trisignata hellenica*
East European	*Cephalota elegans stigmatohora*
West Palaearctic	*Cicindela campestris campestris*, *Cylindera germanica germanica*, *Cicindela hybrida*
Turano-European	*Cicindela monticola rumelica*
South European	*Cicindela sylvicola*, *Cylindera germanica muelleri*
Pannonian-Sarmatian	*Cicindela soluta pannonica*
Euro-Siberian	*Cicindela sylvatica*, *Cylindera arenaria viennensis*
Afrotropico Indo-Mediterranean	*Myriochila melancholica melancholica*

### Distribution of tiger beetles in the Balkans

**Figure 3.**
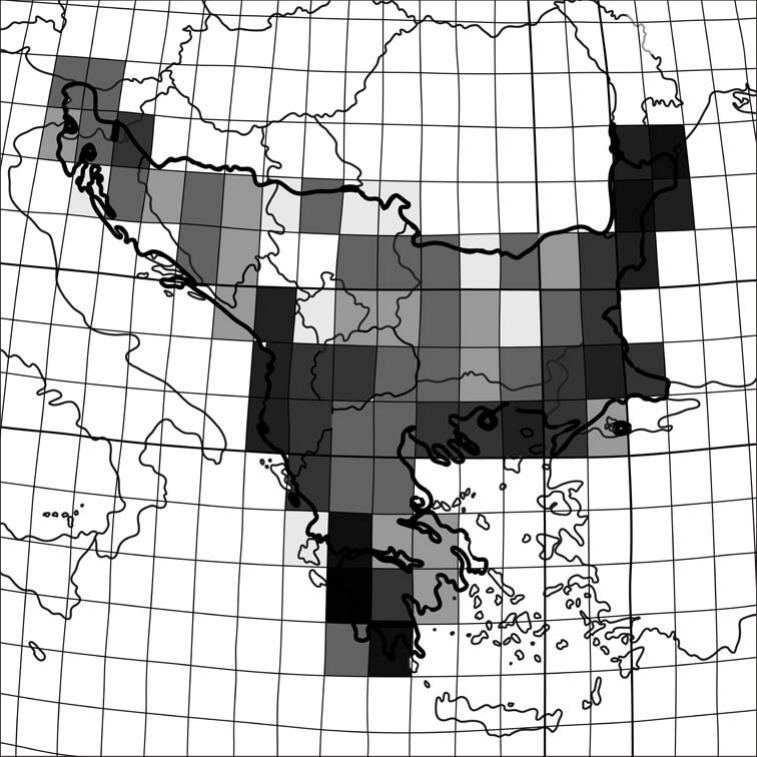
Species richness of tiger beetles within the Balkan Peninsula. The colour gradient indicates an enhanced diversity from one species (white square) to eight (black square).

Within the Balkan Peninsula, species richness of particular regions differs both in number of taxa and species composition. Records from the literature and my own observations within single squares of 1o latitude and longitude show that the highest numbers of tiger beetle species are along sea coasts (Fig. 3). Moreover, within biogeographic provinces as definied by Udvardy (1975) the greatest tiger beetle species richness in the Balkan Peninsula is found in the Mediterranean Sclerophyl region (13 species, 68% of the Balkan fauna), and somewhat lower in the Pontian Steppe (10 species, 52%), and the Balkan Highlands (12 species, 63%). This, despite the fact that the Balkan Highlands cover a part of the peninsula that is larger than both previous biogeographical provinces combined. Moreover, the Balkan part of the Pontian Steppe is almost 17 times smaller than the Balkan Highlands and about ten times smaller than the Mediterranean Sclerophyl (Fig. 2).

**Figure 4.**
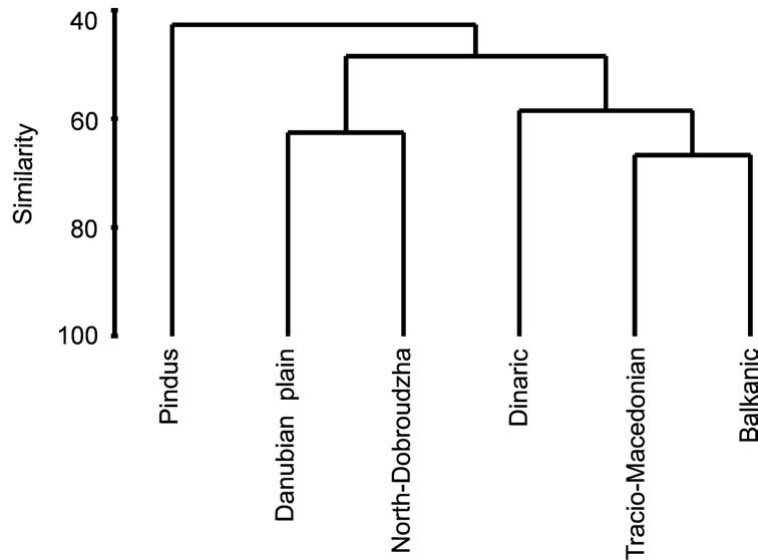
Similarities among tiger beetle faunas inhabiting regions of the Balkan Peninsula (Bray*-*Curtis similarity index for presence*/*absence data).

Bray*-*Curtis analysis of similarities among tiger beetle faunas from different Balkan geographical regions shows the presence of three main clades (Fig. 4). The Dinaric, Tracio-Macedonian and Balkanic regions group mainly mountain areas, with lowlands only as very small parts, and covers a great part of Udvardy’s (1975) Balkan Highlands. The Danubian plain and North-Dobroudzha regions compose the second group, mentioned in biogeographic studies as the Pontian Steppe and north-eastern part of the Balkan Highlands. Clearly different is the Pindus area, which covers a large area of the Mediterranean Sclerophyl province. The Jaccard’s similarity index for Mediterranean Sclerophyl – Pontian Steppe was 53%, for Mediterranean Sclerophyl – Balkan Highland was 47%, and for Pontian Steppe – Balkan Highland was 38% (Fig. 4).

### Ecotypes of Balkan Cicindelidae

The most eurytopic species are *Calomera littoralis nemoralis* and *Calomera aulica aulica* (Table 3), occupying four and three habitats respectively. Ten species have been found occurring in only one type of habitat, including three *Cephalota* species in saltmarshes, three species restricted to sandy sea coasts (*Calomera concolor*, *Cylindera contorta*, *Cylindera trisignata*), four to river banks (*Calomera fischeri*, *Cicindela sahlbergii albanica*, *Cylindera soluta pannonica*, *Cylindera arenaria viennensis*), and one – *Cicindela sylvatica*, to forested sandy areas. Another five species were noted as occurring only in two types of habitat. Among all these tiger beetles, fifteen species (79% of the fauna) can be classified as coastal and riverine taxa, occurring in habitats adjacent to water, such as sea coasts, salt marshes (including lagoons and estuaries), and banks of rivers and freshwater lakes.

**Table 3.** Tiger beetles of the Balkan Peninsula and their ecological distribution: **1** salt marshes **2** sandy sea beaches **3** banks of rivers **4** banks of lakes **5** forest roads **6** mountain and highland pastures **7** flat coastal rocks (based on literature data and personal observations).

*No.*	*Species*	*1*	*2*	*3*	*4*	*5*	*6*	*7*
1	*Calomera aulica aulica*	+	+					+
2	*Calomera concolor concolor*		+					
3	*Calomera fischeri fischeri*			+				
4	*Calomera littoralis nemoralis*	+	+	+	+			
5	*Cephalota (Cephalota) turcica*	+						
6	*Cephalota (Taenidia) chiloleuca*	+						
7	*Cephalota (Taenidia) circumdata circumdata*	+						
8	*Cephalota (Taenidia) elegans stigmatophora*	+		+				
9	*Cicindela (Cicindela) campestris*					+	+	
10	*Cicindela (Cicindela) hybrida*			+			+	
11	*Cicindela (Cicindela) monticola albanica*			+				
12	*Cicindela (Cicindela) soluta pannonica*			+				
13	*Cicindela (Cicindela) sylvatica*					+		
14	*Cicindela (Cicindela) sylvicola*					+	+	
15	*Cylindera (Cylindera) germanica*	+					+	
16	*Cylindera (Eugrapha) arenaria viennensis*			+				
17	*Cylindera (Eugrapha) contorta contorta*		+					
18	*Cylindera (Eugrapha) trisignata*	+	+					
19	*Myriochila (Myriochila) melancholica melancholica*	+		+				
Total		9	5	8	1	3	4	1

## Discussion and conclusions

### Diversity and distribution of tiger beetles in the Balkan Peninsula

Compared to the area size of other regions of Europe, the diversity of tiger beetles of the Balkan Peninsula is high and constitutes about 41% of all European tiger beetle species. This result confirms an important role of the Balkans as a biodiversity hotspot noted earlier for many other groups of organisms (Blonden and Aronson 1999, Kryštufek and Reed 2004, Thompson 2005). The high diversity of tiger beetles in the Balkans can be explained by two characteristics. The first is the topographic position of this area within the European continent – the Peninsula was (and still is) a natural dispersal bridge for faunas from the Middle East and West, North and East Europe. The second is the high diversity of open habitats prefered by these beetles, including salt marshes, salty lagoons, sandy beaches, river banks, steppes, or mountain areas.

The Balkan Peninsula is inhabited by a mixed tiger beetle fauna with representatives of 19 species belonging to 14 different groups according to their geographical origin (Table 2). Such a mosaic of faunal elements clearly suggests an important role of the Balkan Peninsula as a natural geographic „bridge” between Europe and Asia Minor for this group in the past. Similar patterns have been noted also among other groups of insects (Kenyeres et al. 2009), spiders (Deltshev 1999, 2000, 2004), amphibians and reptiles (Crnobrnja-Isailovic 2007; Džukić and Kalezić 2004), mammals (Kryštufek 2004) and plants (Thompson 2005).

A high level of landscape heterogeneity also helps in explaining the general distribution pattern of tiger beetle species within the Balkan Peninsula and their higher species richness in the lowlands. Sandy habitats preferred both by larvae and adult are more diverse at sea coasts than those found in mountain areas. This patterns for Balkan tiger beetles is similar to that reported from other regions of the Mediterranean area (Cassola 1970, 1973, Lisa 2002, Jaskuła – unpublished). Moreover, a higher diversity of tiger beetles along sea coasts over that found in mountain areas has been found on the Indian subcontinent and in western and northern Australia (Pearson and Cassola 1992). It is most likely attributed to high habitat diversity occurring in lower altitudes (sandy beaches, salt marshes, lagoons, dunes, ect).

**Plate 1.**
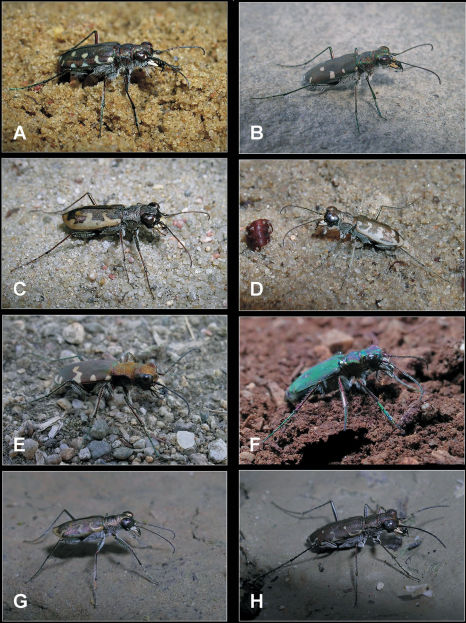
Balkan tiger beetle species: **A** *Calomera littoralis nemoralis* **B** *Calomera fischeri fischeri* **C** *Cephalota chiloleuca* **D** *Cephalota chiloleuca circumdata* **E** *Cicindela sylvicola* **F** *Cicindela campestris oliviera* **G** *Cylindera trisignata hellenica* **H** *Myriochila melancholica melancholica*.

### Ecological preferences in Balkan Cicindelidae

The narrow specialization to habitat type recorded for most of the Balkan tiger beetle species is similar to that in tiger beetles occurring in other regions of the world, both for adults and larvae. For example, of the 151 species noted on the Indian subcontinent by Acciavatti and Pearson (1989) only one – *Calochroa flavomaculata* Hope – was recorded from several different habitat types. In Australia among 29 species only two – *Myriochila mastersi* Castelnau and *Myriochila semicincta* Brulle – occur found as occurring in several habitat types (Freitag 1979). In the Tambopata Reserve Zone (Madre de Dios, Peru) only one of 29 species – *Odontocheila annulicornis* Brulle – occurred in more than one forest habitat type (Pearson 1984), and of the 20 species noted in the Sulphur Springs Valley (Arizona, USA) only *Cicindelidia nigrocoerulea* Leconte was recorded as inhabiting more than one habitat type (Knisley and Pearson 1984). Moreover, the specialization can be so narrow that species occurrence can be restricted to only a small part of a particular habitat. Schultz and Hadley (1987) showed during their studies of two riparian species in the USA that *Cicindela oregona* Leconte occurred mainly at stream edges while *Cicindela tranquebarica* (Herbst) preferred dry areas. Also Ganeshaiah and Belavadi (1986) noted that four tiger beetle species segregated distinctly along river beds into separate microhabitats in India. In the Balkans, I observed similarly narrow microhabitat specialization in the Evros river delta (eastern Greece) for *Calomera littoralis nemoralis* (wet sand), *Cephalota circumdata circumdata*, *Cylindera trisignata hellenica* (dry parts of river bed), and in the Danube river delta (eastern Romania) for *Cephalota chiloleuca* (drier salt marsh substrate), and *Calomera littoralis nemoralis* (edge of reservoirs).

Such narrow specialization to habitat/microhabitat types among tiger beetle species is explained by physiological (Schultz and Hadley 1987, Hadley et al. 1990), morphological (Pearson and Mury 1979, Schultz and Hadley 1987), and behavioural (Knisley and Pearson 1981, Pearson and Lederhouse 1987) adaptations of adults and larvae.

Most Balkan tiger beetles occupy sandy habitats localized in lowlands, mainly on the sea coasts and in river deltas (Table 3). More than 90% of south-east European salt marshes are found in the Balkan Peninsula (Dijkema 1984). As a result of human activity some of these areas have been significantly altered (Saveljić 2008, Davy et al. 2009) and are threatened. Therefore, this habitat is included among important biodiversity sites in the European Union’s Habitats Directiveand Water Framework Directive (Directive 1992, 2000). Given the ecological distribution of Balkan tiger beetles (Table 3), at least 42% of the recorded species occur in these threatened environments. Moreover, studies of *Calomera* species show that some tiger beetles characteristic of coastal sandy beaches are negatively influenced by tourist activity and rapid development of tourist infrastructure (Arndt et al. 2005). If valid for the Balkan Peninsula, this adds an additional two or three species to the list of potentially threatened tiger beetles, and a total of almost 60% of all Balkan tiger beetle fauna. What more, the Balkan Peninsula is a biogeographical melting pot, and a transition zone where faunal elements of various origins meet. Thus, such a biogeographical structure, unique both at a scale of the southeastern Mediterranean region and the entire European continent, is particularly vulnerable to deterioration.

Hopefully the plight of these tiger beetles will help focus the attention of biologists, ecologists, and nature conservationists on the Balkan Peninsula as an important European hotspot area for conserving biodiversity of the European fauna.
